# Cutaneous adipose tissue carries a strong inflammatory signature in patients with psoriasis

**DOI:** 10.1172/jci.insight.194171

**Published:** 2026-03-03

**Authors:** Naomi Shishido-Takahashi, Sandra Garcet, Inna Cueto, Hong Beom Hur, Elisa Muscianisi, Jennifer Steadman, Andrew Blauvelt, James G. Krueger

**Affiliations:** 1 Laboratory for Investigative Dermatology, and; 2 Research Bioinformatics, Center for Clinical and Translational Science, The Rockefeller University, New York, New York, USA.; 3 Novartis, Portland, Oregon, USA.; 4 Blauvelt Consulting, LLC, Annapolis, Maryland, USA.

**Keywords:** Dermatology, Immunology, Inflammation, Adipose tissue, Autoimmune diseases, Transcriptomics

## Abstract

This study provides a comprehensive evaluation of the cutaneous adipose tissue (CAT) transcriptome in patients with psoriasis and investigates the effects of IL-17 blockade on CAT inflammation through a randomized placebo-controlled trial using secukinumab (ObePso-S study, ClinicalTrials.gov NCT03055494). RNA sequencing analysis of CAT biopsies from 82 patients with psoriasis revealed 2132 differentially expressed transcripts compared with healthy controls. Notably, significant gene dysregulation was observed in both lesional skin (LS)-CAT and non-lesional (NL)-CAT, including activation of IL-17–driven pathways, antimicrobial peptide–related, and neutrophil degranulation signatures. Stratification by obesity demonstrated that obese psoriatic CAT exhibited a more than 2-fold higher number of differentially expressed genes than non-obese counterparts, suggesting a synergistic interaction between psoriasis and obesity in driving CAT inflammation. Treatment with secukinumab markedly improved inflammatory signatures in psoriatic CAT, with greater improvements observed in obese patients. These findings reveal a pronounced and partially IL-17–dependent inflammatory phenotype in psoriatic CAT, challenge the conventional concept of psoriasis as a solely superficial skin disease, and highlight CAT as an important contributor to systemic inflammation in psoriasis.

## Abstract 2

This study reveals a strong inflammatory signature in cutaneous adipose tissue of psoriasis patients, amplified by obesity and partially dependent on IL-17 signaling.

## Introduction

Psoriasis is a common chronic inflammatory skin disease associated with systemic inflammation and various comorbidities, including diabetes mellitus and premature cardiovascular disease (1, 2). Numerous studies have demonstrated a strong link between psoriasis and obesity (3). Patients with psoriasis are more likely to be obese compared with the general population (4), and those with obesity face an increased risk of cardiovascular disease relative to their non-obese counterparts (5). Moreover, increased adipose tissue has been identified as a strong risk factor in the development of psoriasis (6).

Adipose tissue, which forms an uninterrupted layer over the entire body surface, is predominantly composed of subcutaneous fat (80%–90%), with a smaller proportion of visceral fat (10%–20%) (7). This tissue expands in volume with obesity and functions as an active endocrine organ. Adipokines secreted by adipose tissue have been shown to influence not only metabolic and cardiovascular disease but also psoriasis (8–11). Furthermore, adipose tissue secretes inflammatory mediators crucial to psoriasis pathogenesis, such as TNF-α and IL-6 (12, 13). These findings suggest a potential crosstalk between adipose tissue and immune cells, implicating adipose tissue inflammation in the pathogenesis of psoriasis.

Despite the growing evidence supporting the role of adipose tissue in psoriasis, comprehensive molecular assessments of psoriatic adipose tissue have not been performed. To date, only 2 small studies have investigated a limited number of factors in lesional skin (LS) adipose tissue (14, 15). Here, we present a comprehensive analysis of the cutaneous adipose tissue (CAT) transcriptome in both LS and non-lesional skin (NL) from patients with psoriasis and assess the impact of IL-17 pathway blockade using secukinumab. This approach enables investigation into the role of CAT in psoriasis pathology and its modulation by obesity and targeted immunotherapy.

## Results

### Psoriatic CAT showed major gene expression differences compared with normal controls.

The body contains 2 primary adipose tissue depots: visceral adipose tissue and skin-associated adipose tissue, commonly termed subcutaneous adipose tissue. Subcutaneous adipose tissue is further divided into 2 layers: dermal white adipose tissue, which is located between the reticular dermis and deeper dermal layers, and subcutaneous white adipose tissue, which is located deeper in subcutaneous tissues (Supplemental Figure 1A; supplemental material available online with this article; https://doi.org/10.1172/jci.insight.194171DS1) (16). In this study, biopsies were obtained from the adipose tissue below the dermis (Supplemental Figure 1B), which was largely composed of globules of fat. We designate this tissue as CAT for consistency and clarity.

To verify the adipose identity and integrity of the CAT samples, we assessed the expression of fatty acid–binding protein 4 (FABP4) and leptin, 2 genes highly enriched in adipose tissue compared with skin (internal dataset, not shown). As expected, FABP4 and leptin levels in all CAT samples were markedly higher than those typically observed in skin (Supplemental Figure 2, A and B), supporting the adipose origin of the biopsied tissue. Importantly, expression of both FABP4 and leptin showed a strong positive correlation with BMI across controls and psoriasis patients, with correlation coefficients of 0.76 and 0.8, respectively (Supplemental Figure 2, C and D). Together, these findings confirm the adipose nature and high quality of the CAT samples and indicate that they were not contaminated by overlying skin tissue.

**Figure 1:**
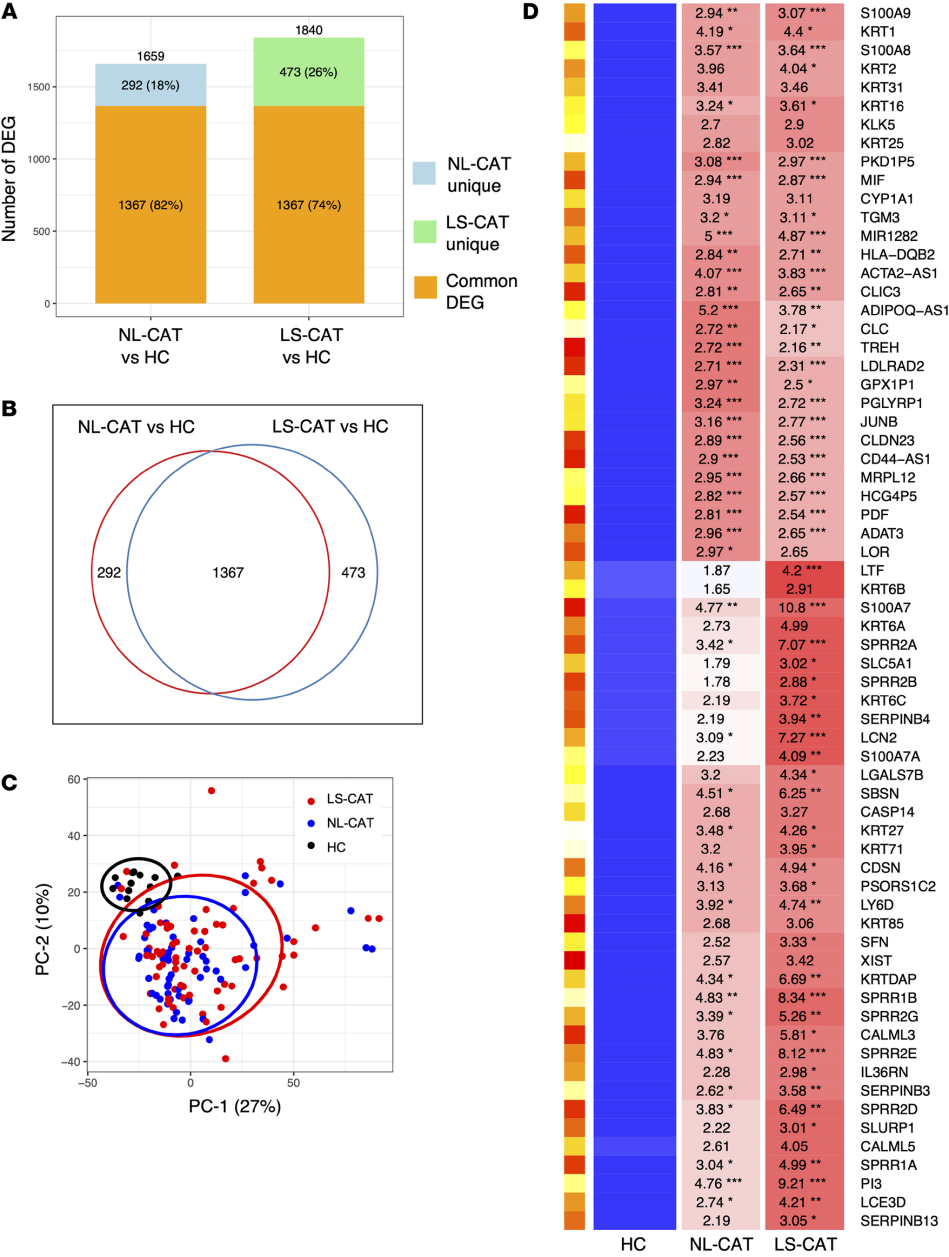
Gene expression differences in LS-CAT and NL-CAT compared with the normal control group. (**A**) The number of DEGs in LS-CAT vs. HC and NL-CAT vs. HC; 1367 DEGs were common to both. (**B**) The psoriasis CAT transcriptome in this study consisted of 2132 transcripts. (**C**) Principal component analysis showed that LS-CAT and NL-CAT overlapped and were separated from the healthy control group. (**D**) Top upregulated genes in CAT transcriptome. DEGs, differentially expressed genes; LS-CAT, lesional skin cutaneous adipose tissue, NL-CAT, non-lesional cutaneous adipose tissue; HC, healthy control. *P < 0.05, **P < 0.01, ***P < 0.001 by limma moderated *t* test.

To identify differentially expressed genes (DEGs), a 1.5-fold change threshold and a false discovery rate of 0.05 were applied to the RNA sequencing data, comparing gene expression in LS-CAT and NL-CAT samples against the control group. In LS-CAT, 741 upregulated and 1099 downregulated genes were identified, while NL-CAT showed 714 upregulated and 945 downregulated genes (Table 1). A total of 1367 genes overlapped between the 2 psoriatic groups (Figure 1A), indicating a substantial shared transcriptomic signature. Together, LS-CAT and NL-CAT accounted for 2132 transcripts when compared against controls (Figure 1B).

Principal component analysis (PCA) demonstrated that the CAT transcriptomes were abundant and distinctly separated from those of healthy controls (Figure 1C), despite a similar distribution of BMI (Table 2). A heatmap of the top upregulated transcripts further highlighted both common and specific changes in gene expression (Figure 1D). Representative psoriasis-related genes included peptidase inhibitor 3 (PI3), S100 family members (including S100A7), adiponectin antisense RNA 1, and neutrophil degranulation–related genes such as lipocalin 2 (LCN2), lactotransferrin (LTF), and macrophage migration inhibitor factor (MIF).

Although LS-CAT and NL-CAT shared a substantial set of DEGs, the heatmap revealed that distinct patterns in the most strongly upregulated transcripts between the 2 groups. Notably, inflammatory gene upregulation was more pronounced in LS-CAT, but NL-CAT also exhibited clear dysregulation of gene expression. Thus, psoriatic CAT from both lesional and non-lesional sites diverges markedly from healthy controls, reflecting a shared core transcriptomic disturbance alongside key differences in the most prominent gene expression changes.

### The gene expression in psoriatic CAT is associated with activation of the IL-17–induced pathway.

**Figure 2:**
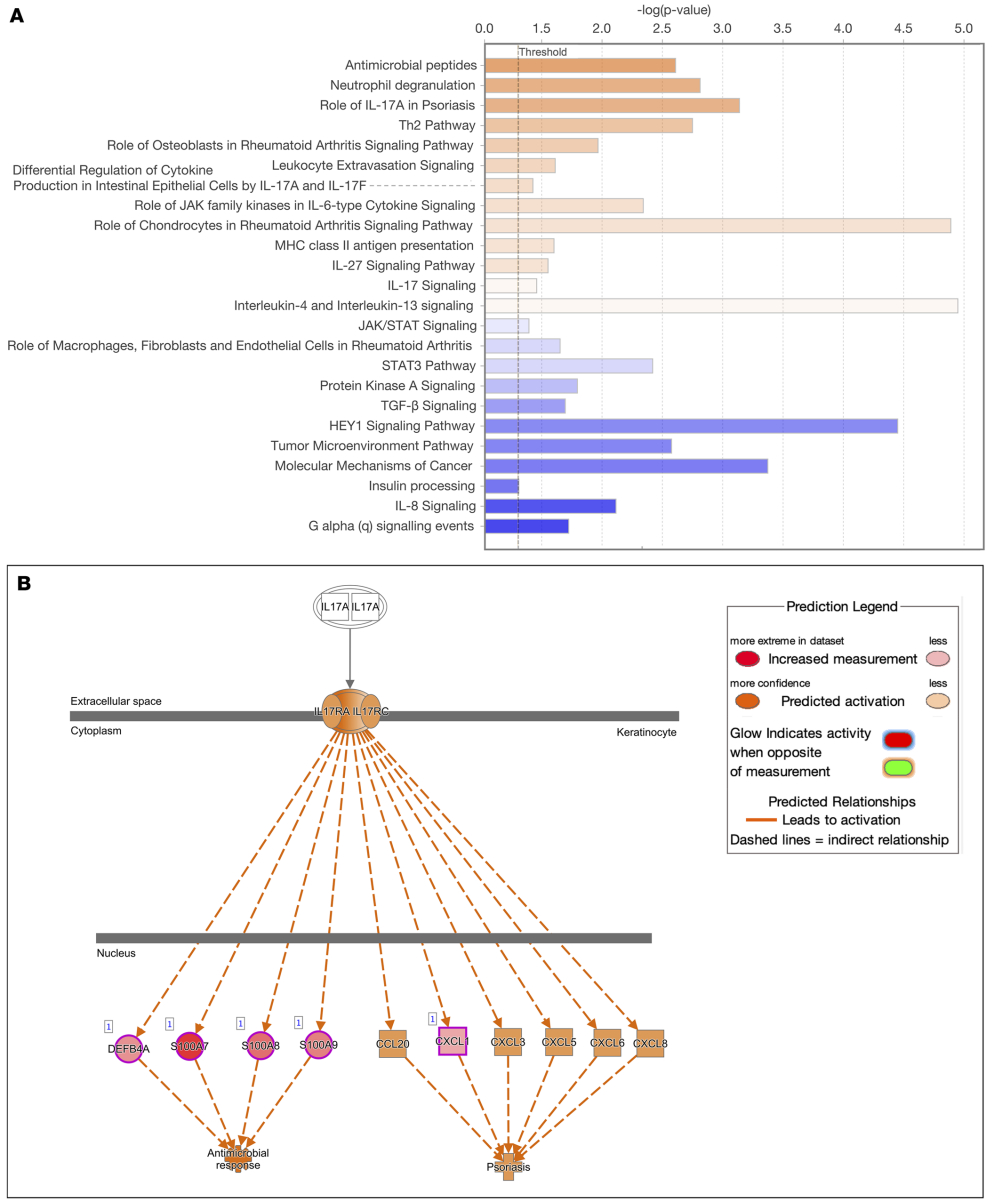
Primary pathways activated or suppressed in psoriatic CAT. (**A**) The top upregulated and downregulated pathways in LS-CAT using Ingenuity Pathway Analysis (*P* < 0.01). The upregulated pathways included several IL-17–related pathways. (**B**) IL-17 signaling pathway in the LS-CAT of patients with psoriasis compared with healthy controls. It was found that IL-17 contributes to the induction of 2 main pathways: an antibacterial response and a psoriasis-inducing response.

To evaluate the biologic significance of gene expression changes identified through transcriptome analysis, a canonical pathway analysis using the Ingenuity Pathway Analysis (IPA) tool was conducted. The top canonical pathways significantly enriched in LS-CAT compared with healthy controls included IL-17A signaling, antimicrobial peptides, and neutrophil degranulation pathways (*P* < 0.01, Figure 2A). IL-17A activity in LS-CAT was associated with 2 primary clusters: one involving the antimicrobial response via activation of defensin β4A (DEFB4A) and the S100 family, and another involving psoriasis-associated chemokines, including CCL20, CXCL1, and CXCL8 (Figure 2B). IL-17 pathway activation was also observed in NL-CAT (data not shown).

### Psoriatic CAT with obesity expresses a wide range of inflammatory genes with potential IL-17 dependency.

Obesity is known to modulate inflammation in adipose tissue of otherwise healthy individuals (17), but relatively little is known about how it impacts psoriatic CAT. To investigate, patients and healthy controls were stratified into obese (body weight ≥ 90 kg) and non-obese (body weight < 90 kg) groups (Table 3).

To further assess whether obesity influenced clustering of transcriptomes, PCA was performed separately for obese and non-obese participants (Supplemental Figure 3). In the non-obese group, baseline LS-CAT and NL-CAT samples clustered closely together, while the non-obese controls formed a distinct and smaller cluster. In the obese group, NL-CAT samples largely overlapped with LS-CAT, whereas obese controls were clearly separated from both psoriasis groups. These results indicate that although obesity amplifies transcriptomic dysregulation in CAT, disease status — rather than weight alone — was the primary driver of separation between psoriasis and control samples.

**Figure 3:**
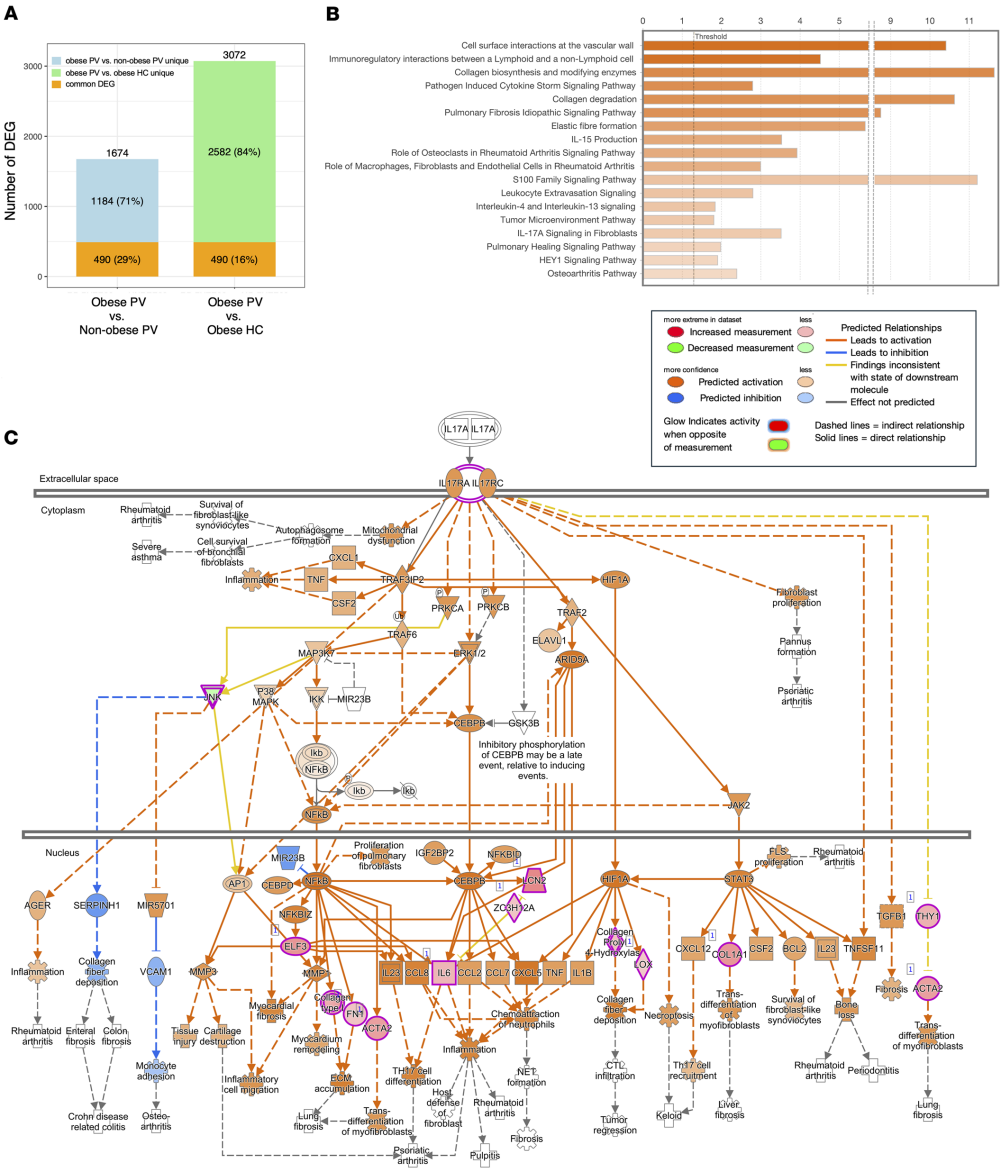
Differential gene expression and activated pathways in obese psoriatic CAT. (**A**) Comparison of the number of DEGs in obese psoriasis patients vs. non-obese patients and vs. obese healthy controls. (**B**) The top activated pathways in LS-CAT of obese patients with psoriasis compared to non-obese patients. It included pathways related to IL-17 signaling. (**C**) IL-17 signaling in LS-CAT in obese patients with psoriasis compared to non-obese patients. PV, psoriasis vulgaris; HC, healthy control.

Quantitatively, obese patients with psoriasis exhibited a substantially larger number of DEGs (3072 genes) compared with obese controls, while non-obese patients with psoriasis showed 1146 DEGs compared with non-obese controls (Table 4). This suggests that obesity expanded DEG expression in psoriasis by more than 100%, indicating an additive or synergistic interaction between psoriasis and adiposity. In the control group, 209 DEGs were observed between obese and non-obese individuals, confirming that obesity affects tissue gene expression. However, when comparing obese patients with psoriasis to obese controls, 3072 DEGs were detected in the psoriatic CAT, which was approximately 15 times higher than in the control group (3072 DEGs and 209 DEGs, respectively). Even within the psoriasis disease spectrum, adiposity influenced gene expression, with 1674 DEGs detected when comparing the CAT of obese and non-obese patients. These results indicate that psoriasis itself increases the expression of DEGs in the CAT of non-obese patients, and this represents a greater effect than the effect of obesity alone (209 DEGs) in the control group. Furthermore, the significantly higher gene expression in obese patients with psoriasis compared with non-obese patients (Figure 3A) suggests an additional interaction between obesity and psoriasis.

Beyond DEG quantity, pathway analysis revealed enrichment of IL-17A, STAT3, and NF-κB signaling pathways, as well as neutrophil mobilization and IL-6/TNF–mediated responses in obese psoriatic CAT (Figure 3, B and C). A heatmap of gene clusters composing these pathways demonstrated consistently higher expression in both LS-CAT and NL-CAT of obese patients compared with non-obese patients (Supplemental Figure 4). Stratifying both psoriasis and control groups by body weight provided further insight. The obese psoriasis group, when compared with obese healthy controls, showed the greatest enrichment in pathways such as metabolism of steroid hormones and leukocyte extravasation signaling. In contrast, the non-obese psoriasis group compared with non-obese healthy controls displayed highest enrichment in neutrophil degranulation and interferon signaling pathways (Supplemental Figures 5–7). These results highlight both shared and divergent pathway regulation as a function of obesity status in both populations. Altogether, these findings indicate that obesity enhances IL-17–driven inflammatory signatures in psoriatic CAT, not only by increasing DEG quantity but also by amplifying expression of key inflammatory mediators and by distinctly influencing the involved biological pathways in psoriasis compared with healthy controls.

### Sex-specific differences in psoriatic CAT gene expression.

Because both obesity and sex are known modifiers of inflammatory processes, we next examined whether transcriptomic changes in psoriatic CAT differed by biological sex. When comparing male and female psoriasis patients separately against controls, the total numbers of DEGs in LS-CAT and NL-CAT were broadly similar, indicating that the overall degree of transcriptomic dysregulation did not differ substantially by sex (Table 5). However, direct comparison of male versus female psoriasis CAT revealed distinct differences in the most highly upregulated genes (Supplemental Figure 8). These contrasts were particularly evident in the non-obese subgroup. For example, S100A7 expression was markedly higher in non-obese male LS-CAT (heatmap value, 20.89) compared with non-obese female LS-CAT (heatmap value, 2.73). Conversely, XIST, a long noncoding RNA mediating X chromosome inactivation, was expressed at levels approximately 20 to 300 times higher in female patients, consistent with its known biological role.

Taken together, these findings indicate that while men and women share a largely overlapping set of DEGs in psoriatic CAT, the regulation of select transcripts differs markedly by sex. These results highlight sex-specific influences on gene expression in psoriatic CAT and suggest potential implications for disease heterogeneity and therapeutic response.

### IL-17A blockade markedly improved inflammation in psoriatic CAT.

**Figure 4:**
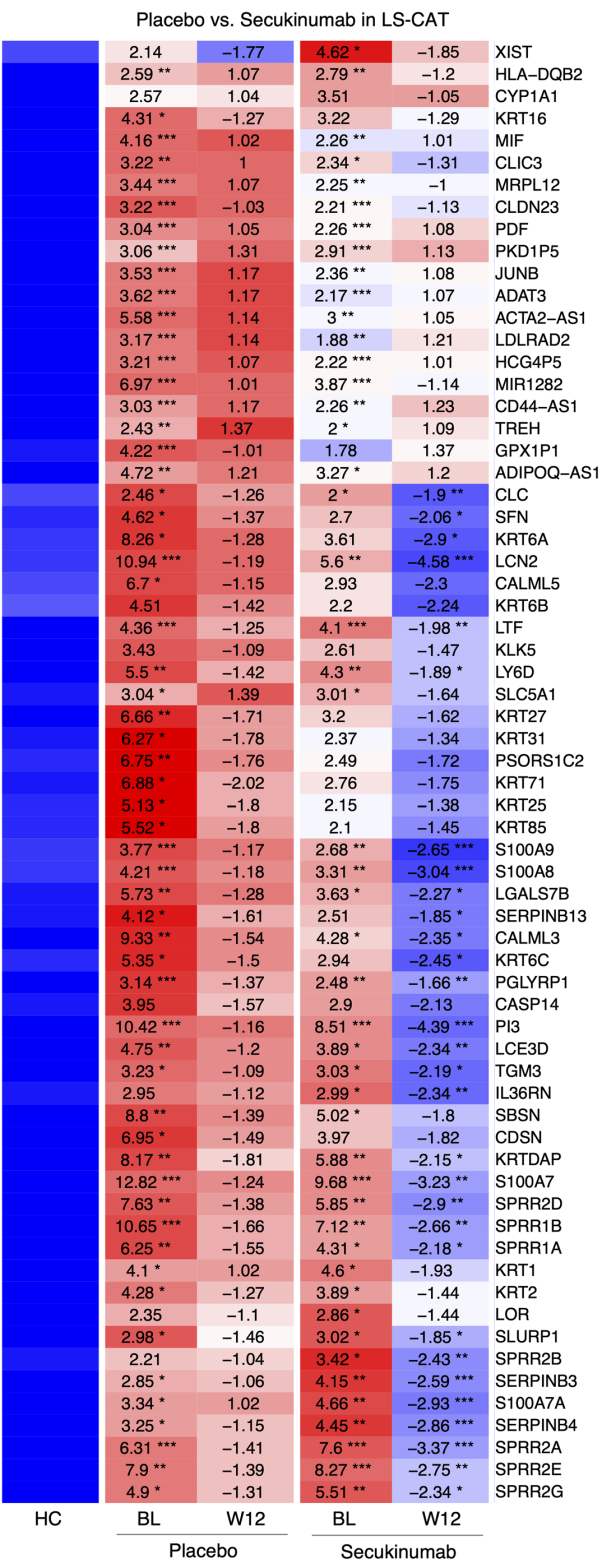
The top upregulated transcripts of LS-CAT in the placebo vs. secukinumab treatment groups. Significant improvements were observed in many genes in the secukinumab treatment group. BL, baseline; W12, week 12; LS-CAT, lesional skin cutaneous adipose tissue. *P < 0.05, **P < 0.01, ***P < 0.001 by limma moderated *t* test.

Analysis of psoriasis CAT suggested that inflammatory genes could be IL-17 dependent. Next, the effects of IL-17 signaling pathway blockade in CAT after 12 weeks of secukinumab treatment were examined. The top upregulated transcripts in LS-CAT showed significant improvement with secukinumab treatment compared with the placebo group (Figure 4). While most highly expressed genes in psoriatic CAT exhibited significant downregulation, some gene expression abnormalities remained after 12 weeks of treatment. Treatment effects were also observed in NL-CAT, with significant improvements in top highly expressed genes, including PI3 and LCN2 (Supplemental Figure 9).

Among the highly expressed transcripts in LS-CAT, proprotein convertase subtilisin/kexin type 9 (PCSK9) ranked within the top 100 genes (Supplemental Figure 10). PCSK9 expression was significantly elevated in psoriatic CAT compared with controls and showed a marked reduction following secukinumab treatment. Comparison of results by body weight revealed that obese patients showed greater improvement with secukinumab treatment than non-obese patients, suggesting enhanced benefits from IL-17 blockade in obese individuals. PI3 and S100 family genes showed meaningful improvement in both obese and non-obese patients, while some genes such as IL36RN and LTF were significantly decreased only in obese patients. Pathway analysis of the CAT transcriptome after treatment demonstrated significant improvement in the IL-17 pathway for both obese and non-obese patients with psoriasis (Supplemental Figures 11 and 12). The neutrophil degranulation pathway showed the most substantial improvement in both groups (*P* < 1 × 10^–10^).

### Analysis of CAT response to IL-17 blockade by level of PASI response.

Patients treated with secukinumab were then stratified into psoriasis area and severity index (PASI) 90 responders (PASI 90R) and PASI 90 non-responders (PASI 90NR) based on PASI on week 12 to compare treatment efficacy at the adipose tissue level. The number of genes showing substantial improvement after treatment was 947 in the PASI 90R group and 1271 in the PASI 90NR group, with the PASI 90NR group exhibiting slightly higher results (Table 6). Genes playing crucial roles in psoriasis pathogenesis, such as PI3, IL36G, and DEFB4A, demonstrated higher improvement rates in the PASI 90R group compared with the PASI 90NR group (Supplemental Figures 13 and 14). Conversely, neutrophil-related genes (LCN2, LTF) showed significant improvement in both the PASI 90R and PASI 90NR groups. These results indicate that both the PASI 90R and PASI 90NR groups benefit from IL-17 blockade in terms of CAT gene expression, suggesting that improvements in CAT inflammation may precede or occur independently of visible skin improvements.

## Discussion

The role of adipose tissue in the pathophysiology of psoriasis has recently emerged as an important area of investigation. Prior studies have reported increased expression of specific gene products in adipose tissue of psoriatic lesions. Okinaga et al. compared the mRNA expression of several genes in psoriatic adipose tissue with those in the skin using qRT-PCR (14). The imaging study by Hjuler et al. demonstrated significantly increased inflammation in adipose tissue of patients with moderate-to-severe psoriasis compared with healthy controls (18). However, a comprehensive molecular evaluation of adipose tissue, including NL areas in patients with psoriasis, has not been conducted to date. This study aimed to analyze the transcriptome of LS-CAT and NL-CAT in patients with psoriasis and evaluate gene expression changes following IL-17A blockade with secukinumab treatment.

Transcriptomic profiling revealed extensive gene dysregulation in psoriatic CAT, with approximately 2000 DEGs in LS-CAT compared with healthy controls. Key psoriasis-related genes, such as PI3 and LCN2, were significantly upregulated. Among the dysregulated gene families, we observed marked upregulation of several S100 family members, including S100A7 (psoriasin), which are well-established contributors to inflammation in psoriasis. S100 proteins act as antimicrobial peptides and proinflammatory alarmins that amplify immune responses through interactions with pattern-recognition receptors such as the receptor for advanced glycation end products and Toll-like receptor 4 (19). Their overexpression has been consistently linked to keratinocyte activation, neutrophil recruitment, and maintenance of chronic psoriatic inflammation. The strong induction of S100 transcripts in CAT highlights that this tissue, like psoriatic skin, participates in the IL-17–driven inflammatory cascade.

Notably, NL-CAT still exhibited approximately 1600 DEGs, and qualitative similarities were also observed between LS-CAT and NL-CAT. These results differ substantially from findings in “regular” skin samples, which consist of only epidermis and dermis. In previous analysis of skin from the same patient cohort, NL skin showed only 5% as many DEGs as LS, compared with healthy controls (20). The findings from psoriatic CAT in the current study underscore the concept that psoriasis is a systemic inflammatory disease extending beyond the cutaneous barrier, highlighting the contribution of CAT to systemic inflammation.

Sex-specific differences were also observed in psoriatic CAT. While the total DEG burden was similar in males and females, several individual transcripts showed pronounced sex-dependent regulation. For instance, S100A7 was strongly induced in non-obese male LS-CAT but expressed at substantially lower levels in female LS-CAT, whereas XIST was expressed at far higher levels in female compared with male LS-CAT. These sex-linked differences are consistent with known biology, such as XIST-mediated X chromosome inactivation, and with recent reports of elevated XIST expression in female psoriasis patients (21). They imply that psoriasis-associated inflammation in CAT may be modulated by sex-specific genetic programs. This observation could help explain differences in clinical presentation, comorbidities, or therapeutic response between male and female patients, and merits further investigation in larger cohorts.

Stratification of study participants — including both patients with psoriasis and healthy controls — by body weight revealed several key biological insights. The comparison between non-obese patients with psoriasis and non-obese healthy individuals revealed a higher number of DEGs than the comparison between obese and non-obese individuals in the control group. This finding indicates that psoriasis itself has a greater influence on DEG expression in CAT than obesity alone. Furthermore, the comparison between obese patients with psoriasis and obese healthy individuals identified over 3000 DEGs, greatly exceeding the effects of obesity or psoriasis alone. These results suggest an additive or synergistic interaction between psoriasis and obesity.

Beyond DEG counts, examination of the top regulated transcripts revealed significant upregulation of MIF, S100 family members (e.g., S100A7), LCN2, and LTF in obese psoriatic CAT compared with controls. These genes play key roles in keratinocyte activation, IL-17–driven inflammation, and neutrophil recruitment, emphasizing mechanistic links between CAT inflammation and systemic psoriasis pathology. Pathway analysis confirmed stronger enrichment of IL-17, STAT3, and NF-κB signaling networks in obese psoriatic CAT, suggesting that obesity magnifies psoriatic inflammation not only quantitatively but also qualitatively through amplification of proinflammatory cascades.In addition to these findings, we observed that IL36RN expression was reduced in obese psoriasis patients after secukinumab therapy. This observation is noteworthy, given that IL36RN typically functions as an antagonist of IL-36 signaling and genetic deficiency in IL36RN has been implicated in generalized pustular psoriasis (GPP) (22). However, the decrease in IL36RN seen here appears to be a pharmacologically induced and reversible effect, distinct from the loss-of-function mutations that predispose to GPP. Moreover, there is no current evidence that IL-17A inhibitors, including secukinumab, increase the risk of GPP flares in obese patients with psoriasis. Still, given the growing clinical relevance of IL-36 biology and the availability of IL-36R inhibitors such as spesolimab for GPP, our results underscore the importance of continued vigilance and mechanistic investigation into IL-36 signaling in psoriatic comorbidities.

We view that virtually all CAT in patients with psoriasis exhibits a distinct disease phenotype compared with healthy controls, including obese individuals without psoriasis. We believe that this is not only a new finding, but also contains a very important message about the disease of psoriasis. CAT has not received much attention in the pathogenesis of psoriasis, but in fact, CAT accounts for a much larger proportion of the human body mass than the epidermis and dermis, with CAT accounting for 25%–36% (23–25) compared with 7%–15% (16, 26) for the epidermis and dermis. As the average body weight of the patients with psoriasis who participated in this study was approximately 100 kg, we consider that at least about 25 kg of CAT, and in obese patients, about 36 kg of CAT, is diseased with high inflammatory gene expression bearing strong psoriasis hallmarks. Even if one considers that there is an inflammatory signal in blood, which many inflammatory cytokines are elevated, the weight is only about 5 kg (5 liters), so this would mean the largest amount of psoriatic tissue in a human is inflamed CAT in the periphery.

IL-17 pathway blockade with secukinumab markedly improved gene expression abnormalities in CAT. This finding provides evidence that IL-17 inhibitors affect gene expression not only in the skin (20), but also in CAT. However, the inflammatory signature in CAT was not completely reversed, implying the involvement of other factors in the co-pathogenesis of the CAT, in addition to IL-17A and this cytokine response axis. In addition to broad transcriptomic improvements, we observed significant downregulation of PCSK9 in psoriatic CAT after secukinumab treatment. PCSK9, a key regulator of lipid metabolism, has also been linked to systemic inflammation and cardiovascular risk, including endothelial dysfunction and increased coronary artery calcium burden in patients with psoriasis. Thus, reduced PCSK9 transcript levels in CAT raise the possibility that IL-17 blockade may beneficially modulate cardiometabolic pathways at the tissue level, even though cardiovascular outcomes were not directly assessed in this study. This finding supports the concept that inflamed CAT may contribute to systemic comorbidity and identifies PCSK9 as a potential mechanistic connector between skin inflammation and cardiovascular disease in psoriasis. These findings should, however, be interpreted with caution. A major limitation is the lack of paired protein measurements in skin, CAT, and blood, which prevents direct confirmation that transcript changes translate into altered PCSK9 protein abundance locally or systemically. Moreover, prior studies have generally reported minimal effects of secukinumab on traditional cardiometabolic risk factors and cardiovascular events, despite robust antiinflammatory activity. Therefore, our PCSK9 results should be regarded as hypothesis-generating rather than definitive evidence of cardiometabolic benefit, and future longitudinal studies integrating proteomics, circulating PCSK9 and lipid markers, and clinical cardiovascular endpoints will be required to clarify their clinical significance.​

Biologic functions of the most responsive genes were associated with the IL-17 pathway and neutrophil degranulation. IL-17 is a crucial factor in promoting neutrophil chemotaxis and inducing neutrophil infiltration into tissues (27). While CD4^+^ Th17 lymphocytes have traditionally been considered the main source of IL-17, recent report suggests that neutrophils may become a source of IL-17A during acute inflammation (28). Furthermore, it has been shown that IL-17 is released during neutrophil extracellular trap formation in the skin of patients with psoriasis (29). Although the source of IL-17A remains debatable, its neutralization has been proven to correlate with decreased levels of neutrophil chemotactic chemokines such as CXCL1 and CXCL2, leading to reduced neutrophil infiltration into tissues (30–32). This suggests that IL-17A is a potent inducer of CXC chemokines and subsequent neutrophil infiltration. While previous studies have not exclusively focused on CAT, they imply that IL-17 plays a crucial role in the recruitment and infiltration of neutrophils across various tissues, including CAT.

Overall, our data demonstrate that CAT exhibits gene dysregulation that is not only limited to LS-CAT but also extends to NL-CAT. These dysregulated genes were partially IL-17 dependent and improved with IL-17 pathway blockade. This study provides insight into the conventional view of psoriasis as a superficial inflammatory disease, and our results suggest that CAT, which has a significant expression of inflammatory genes and a larger proportion of the body than skin or blood, may play a crucial role in the pathogenesis of psoriasis. Future research will need to be longitudinal in order to clarify how inflamed CAT affects systemic inflammation in psoriasis and how these changes contribute to clinical improvement.

An important limitation of this study relates to the composition and size of the control group. The present analysis leverages a preexisting, prospectively recruited clinical cohort that was originally assembled for a skin-focused transcriptomic study, and only a subset of participants had cutaneous adipose biopsies of sufficient quality for RNA sequencing. As a consequence, the available control samples were fewer, older on average, and included only one female, which restricted our ability to perform robust multivariable adjustment or stratified analyses by age, sex, and BMI. Although all psoriasis patients and controls were recruited at the same center under a single protocol and processed using identical laboratory procedures, residual and unmeasured confounding cannot be excluded and may contribute to some of the observed differences between psoriasis and control CAT. For these reasons, our comparisons between patients with psoriasis and controls should be interpreted as exploratory and hypothesis-generating rather than providing fully adjusted estimates of psoriasis-specific effects, and larger, prospectively designed cohorts with better balanced demographic and metabolic characteristics will be required to validate these findings. Another limitation is the use of bulk RNA sequencing, which captures total gene expression from the diverse cell populations present in CAT. This includes not only adipocytes but also infiltrating immune cells, stromal fibroblasts, and endothelial cells, all of which contribute to the observed transcriptomic signatures. Consequently, pathway enrichment results should be interpreted at the tissue level rather than as adipocyte-specific phenomena. For example, the enrichment of IL-17–related signaling and neutrophil degranulation pathways likely reflects contributions from infiltrating immune cells interacting with adipocytes and stromal components in the lesional adipose microenvironment. Nevertheless, our IPA results, including the specific gene sets driving key pathways, offer improved transparency and mechanistic insight by clarifying how composite cell signals shape the observed inflammatory programs. Taken together, these data highlight the complex cellular crosstalk in CAT of patients with psoriasis and provide a framework for future studies employing higher-resolution methods.

## Methods

### Sex and demographic variables as biological factors.

CAT samples from both male and female patients with psoriasis and controls were included for transcriptome analyses (Tables 2 and 7). To evaluate potential confounding by demographic or anthropometric factors, we compared age, sex, race, weight, and BMI between psoriasis and control groups (Table 2). Most variables did not differ significantly; however, controls were on average older and exhibited a sex imbalance. To address these differences, sensitivity analyses by sex were conducted, which revealed no significant impact on transcriptomic outcomes, indicating that the observed CAT expression profiles were not confounded by demographic or anthropometric variables.

### Study design.

This study is a post hoc analysis of a randomized, double-blinded, placebo-controlled phase IV study (ObePso-S study, ClinicalTrials.gov NCT03055494), which was conducted between April 2017 and February 2019 in accordance with the Declaration of Helsinki. Eligible patients with moderate-to-severe plaque psoriasis provided written informed consent prior to study participation. Patient demographics are presented in Table 2. The patients were randomized 2:1 to receive either secukinumab (*n* = 54) or placebo (*n* = 28) for 12 weeks using an Interactive Response Technology system.

The randomization was stratified by body weight (non-obese < 90 kg or obese ≥ 90 kg). To ensure analytic rigor and control for confounding factors, healthy controls were stratified into obese and non-obese groups using the same thresholds applied to patients with psoriasis (Table 3). This allowed direct weight-matched comparison and uncovered baseline and treatment-related transcriptomic differences specific to both disease status and adiposity. Secukinumab was administered according to the approved label (300 mg subcutaneously on weeks 0, 1, 2, 3, and 4, and every 4 weeks thereafter), while patients in the placebo group received matching subcutaneous placebo injections. After the placebo-controlled phase, all patients were re-inducted in a blinded fashion and assigned to treatment with secukinumab. Details of the primary and secondary outcome measures and the results of the study have been reported previously (20).

### Sample biopsy.

CAT biopsy tissue was obtained by performing a second punch biopsy from a deeper part of the same area after a first skin punch biopsy from the epidermis to the adipose tissue (Supplemental Figure 1B). LS-CAT and NL-CAT biopsies were performed at baseline and on week 12 on 82 patients with psoriasis. Control samples were also collected from 15 healthy participants using the same procedure. RNA was isolated from the CAT sample using a RNeasy Lipid Tissue Mini kit (QIAGEN). The consort diagram from patient screening to profiling is shown in Supplemental Figures 15 and 16.

### Pathway analysis.

DEGs identified by limma were further analyzed using IPA (QIAGEN). IPA was used to evaluate canonical pathways, biological functions, and upstream regulators, and to calculate activation *z* scores. To improve transparency, we provide the genes contributing to selected pathways, including IL-17 signaling and neutrophil degranulation, in supplemental figures. These data highlight the specific gene clusters driving enrichment of pathways implicated in psoriasis-associated inflammation. Because bulk RNA sequencing was employed, the resulting transcriptomes represent composite signals derived from adipocytes, immune cells (e.g., neutrophils, macrophages, lymphocytes), stromal fibroblasts, and endothelial cells present in CAT. Thus, pathway enrichment results reflect integrated tissue-level patterns rather than cell-type–specific contributions.

### Statistics.

Principal component analysis (PCA) was conducted as an unsupervised dimensionality reduction approach to visualize the overall data structure, identify potential outliers, and evaluate separation between groups. Statistical analyses were conducted using R software (R Foundation; R-project.org). Gene expression profiles were analyzed using linear mixed models implemented in the limma package (Bioconductor). Fixed effects included treatment (secukinumab vs. placebo), time point (baseline vs. week 12), and tissue type (LS-CAT vs. NL-CAT), while random effects related to each patient were included. The mixed-model estimation and subsequent IPA were also extended to evaluate the effect of body weight (<90 kg vs. ≥90 kg). Least-squares means were calculated for each group, and fold changes were determined for all comparisons. Differential expression between groups was assessed using moderated *t* statistics implemented in the linear modeling framework. Statistical significance was set at a *P* value of less than 0.05. Correlations between fat-related factors (leptin and FABP4) and BMI were analyzed using Pearson’s correlation coefficient (*r*).

### Study approval.

CAT samples were collected from the individuals after the provision of written, informed consent. The study protocol was approved by the central institutional review board and the institutional review boards or independent ethics committees of each participating facility.

### Data availability.

Data are available upon request. All genomic data generated in this manuscript have been deposited in the NCBI’s Gene Expression Omnibus (GEO) database (GSE201827, GSE287022).

## Author contributions

NST and JGK designed the study. AB contributed conceptual ideas. JGK collected clinical data and biospecimens. SG and HBH performed the statistical analyses, and NST and JGK interpreted the results. IC managed data. EM and JS acquired funding. NST generated figures and wrote the manuscript. All authors edited, reviewed, and approved the final manuscript.

## Funding support

- Novartis.
- Japan Society Promotion of Science grant (to NST).

## Supplementary Material

Supplemental data

Supporting data values

## Figures and Tables

**Table 1:**
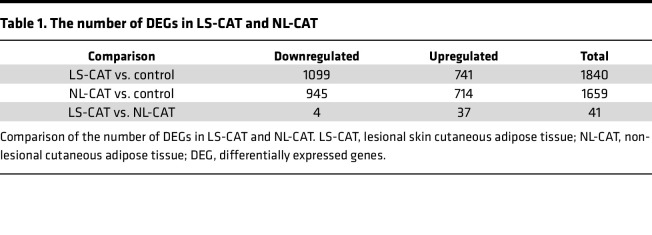
The number of DEGs in LS-CAT and NL-CAT

**Table 2:**
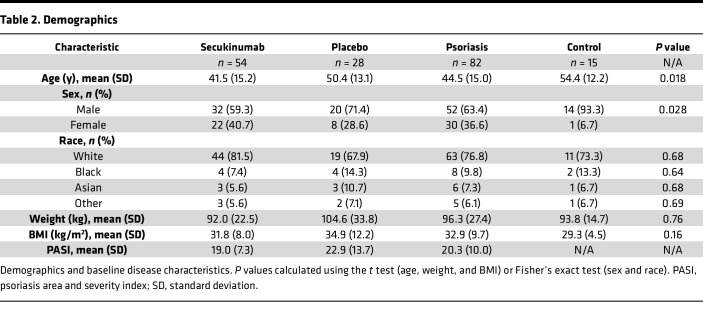
Demographics

**Table 3:**
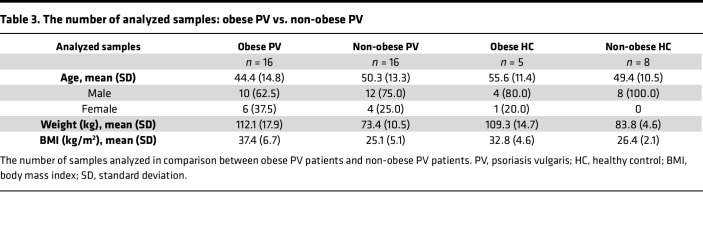
The number of analyzed samples: obese PV vs. non-obese PV

**Table 4:**
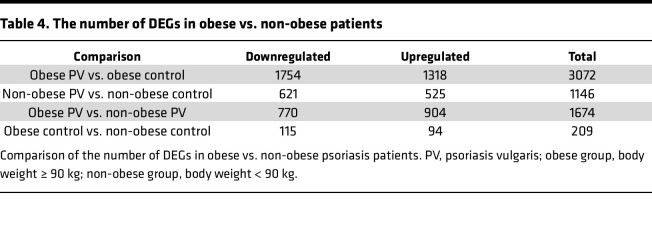
The number of DEGs in obese vs. non-obese patients

**Table 5:**
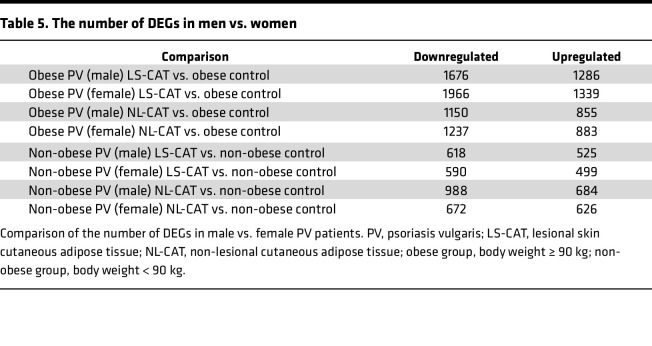
The number of DEGs in men vs. women

**Table 6:**
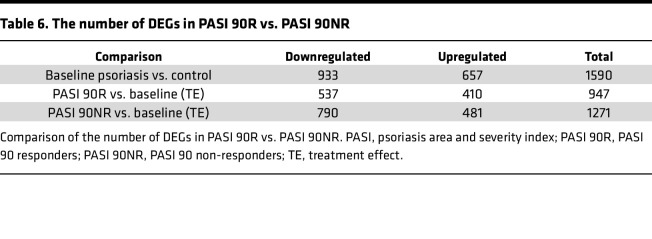
The number of DEGs in PASI 90R vs. PASI 90NR

**Table 7:**
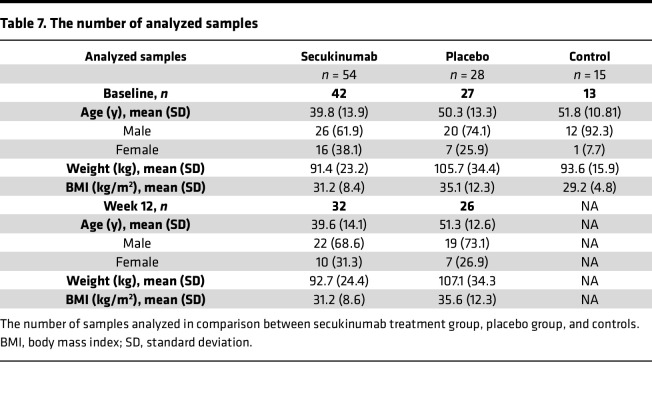
The number of analyzed samples

## Version 1 03/03/2026

In-Press Preview

## Version 2 04/08/2026

Electronic publication
