# Supplementary material for: Cutaneous adipose tissue carries a strong inflammatory signature in patients with psoriasis
Source: JCI Insight. 2026 Mar 3;11(7):e194171. doi: 10.1172/jci.insight.194171 (PMC13134718; doi:10.1172/jci.insight.194171)
Supplement: Supplemental data [file jciinsight-11-194171-s295.pdf]

Supplemental figures

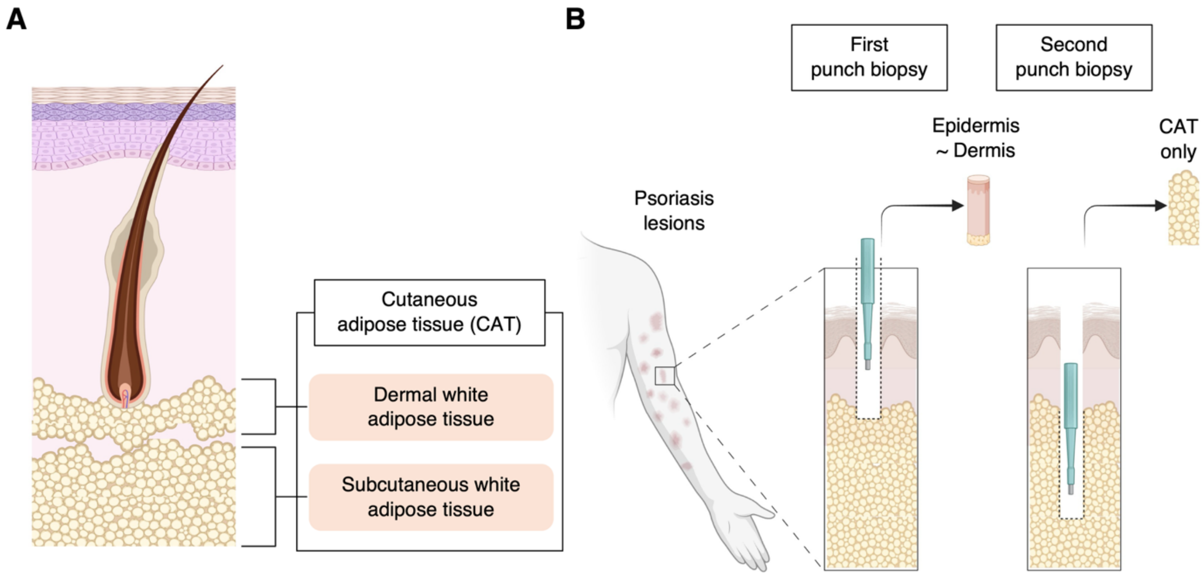

Supplementary Figure 1. (A) Distribution of dermal and subcutaneous white adipose tissues. The cutaneous adipose tissue (CAT) biopsy included both layers. (B) Diagram of CAT tissue biopsy. The biopsy samples were taken using a second deep subcutaneous tissue biopsy.

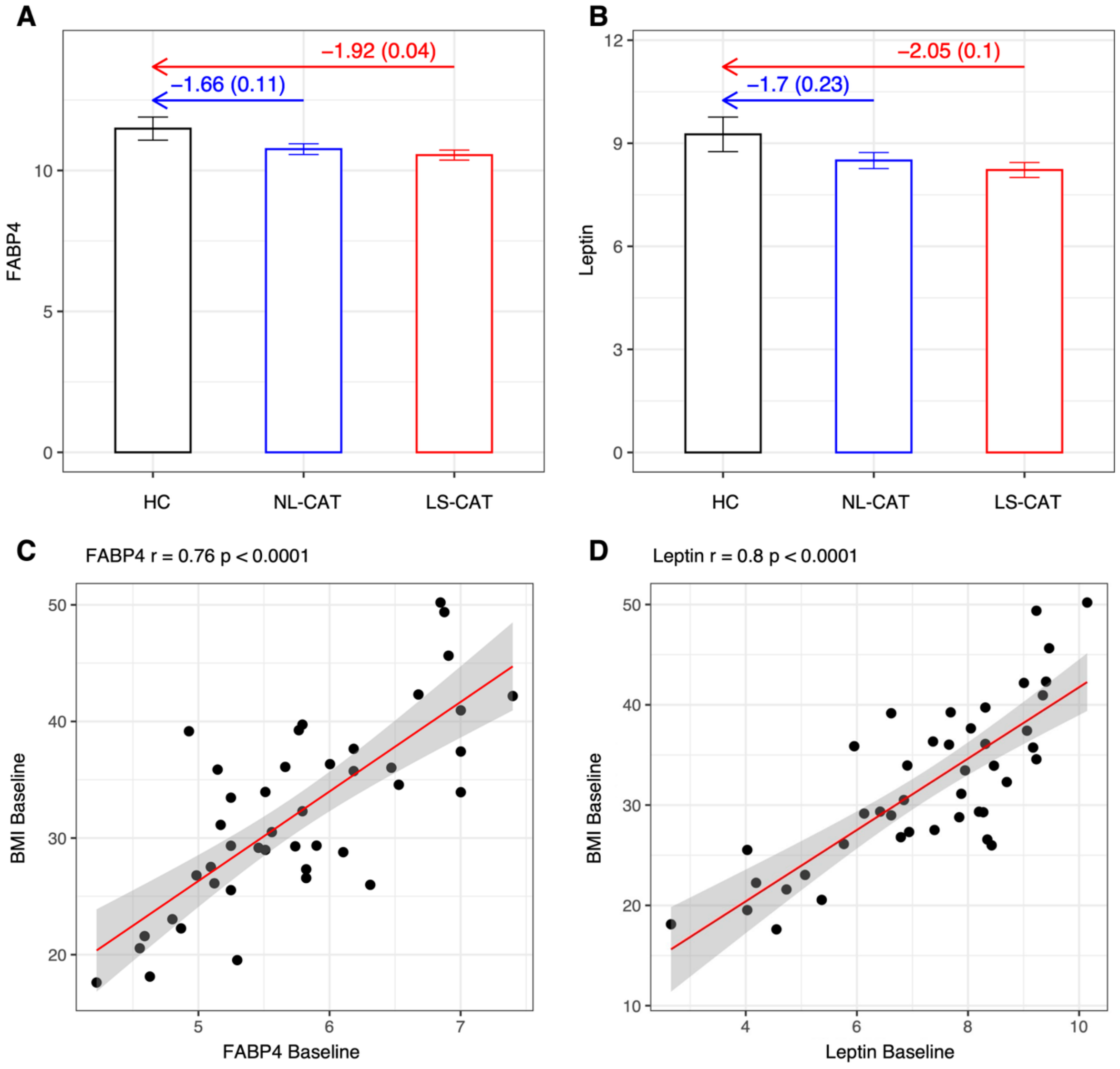

Supplementary Figure 2. Gene expression levels of (A) FABP4 and (B) leptin in CAT. Correlation between BMI and the expression level of (C) FABP4 and (D) leptin at baseline. (A and B) Differential expression between groups was assessed using moderated t-statistics, and P values were adjusted for multiple testing using the Benjamini–Hochberg method. (C and D) r = Pearson correlation coefficient. FABP4, fatty acid binding protein 4; CAT, cutaneous adipose tissue; LS, lesional skin; NL, non-lesional skin; HC, healthy control.

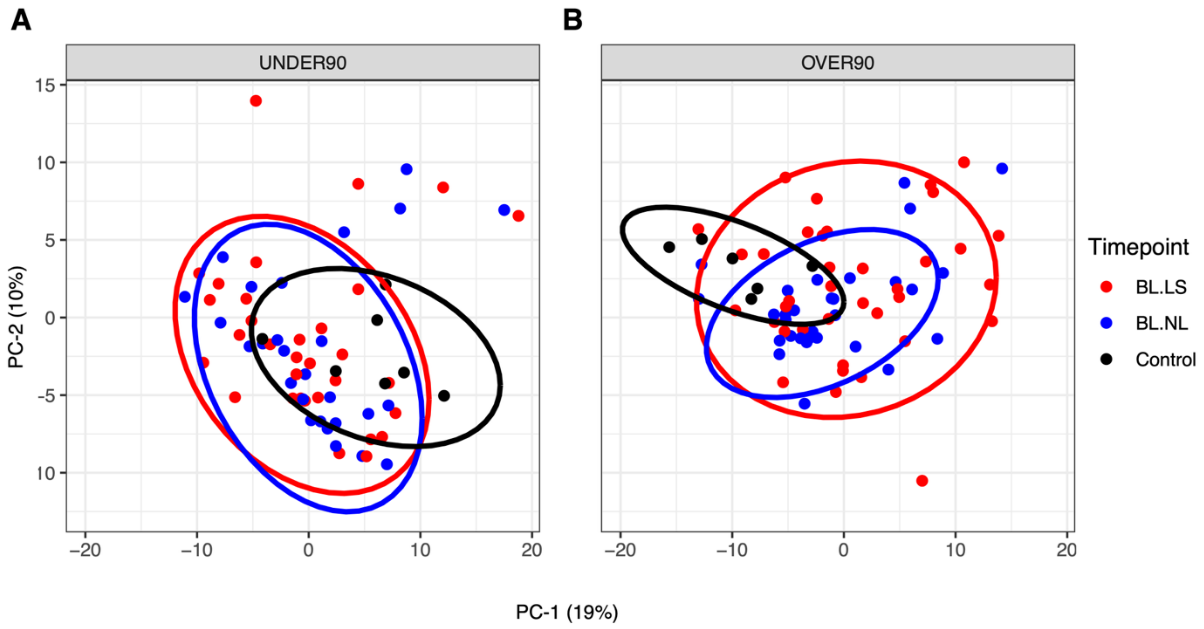

Supplementary Figure 3. Principal component analysis for the obese (OVER90) group and the non-obese (UNDER90) group. BL.LS, base line lesional skin cutaneous adipose tissue; BL.NL, base line non lesional skin cutaneous adipose tissue; Control, control group cutaneous adipose tissue.

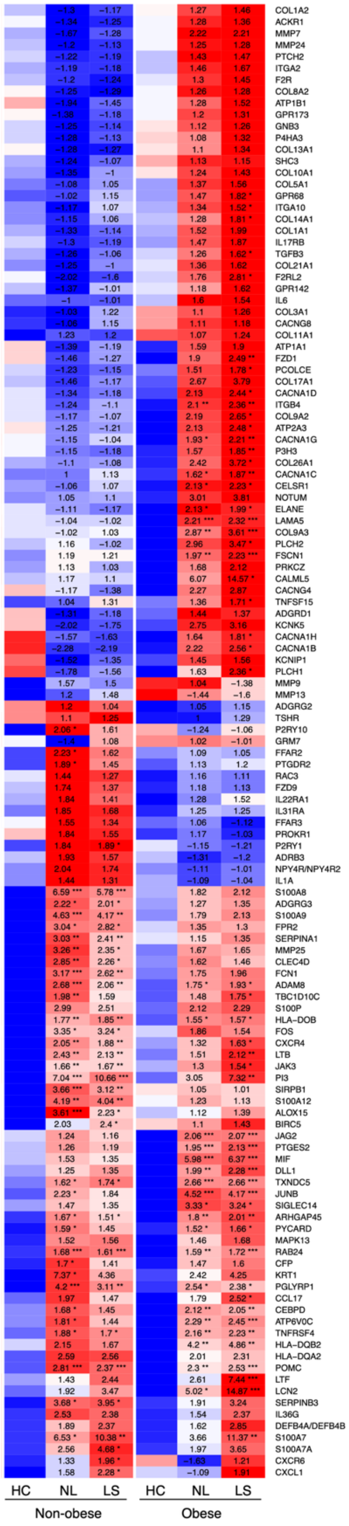

Supplementary Figure 4. The top expressed gene clusters composing the activated pathways in LS-CAT and NL-CAT (Figure 3B). *, **, *** indicate $p < 0.05$, $p < 0.01$, $p < 0.001$, respectively. CAT, cutaneous adipose tissue; LS, lesional skin; NL, non-lesional skin; HC, healthy control.

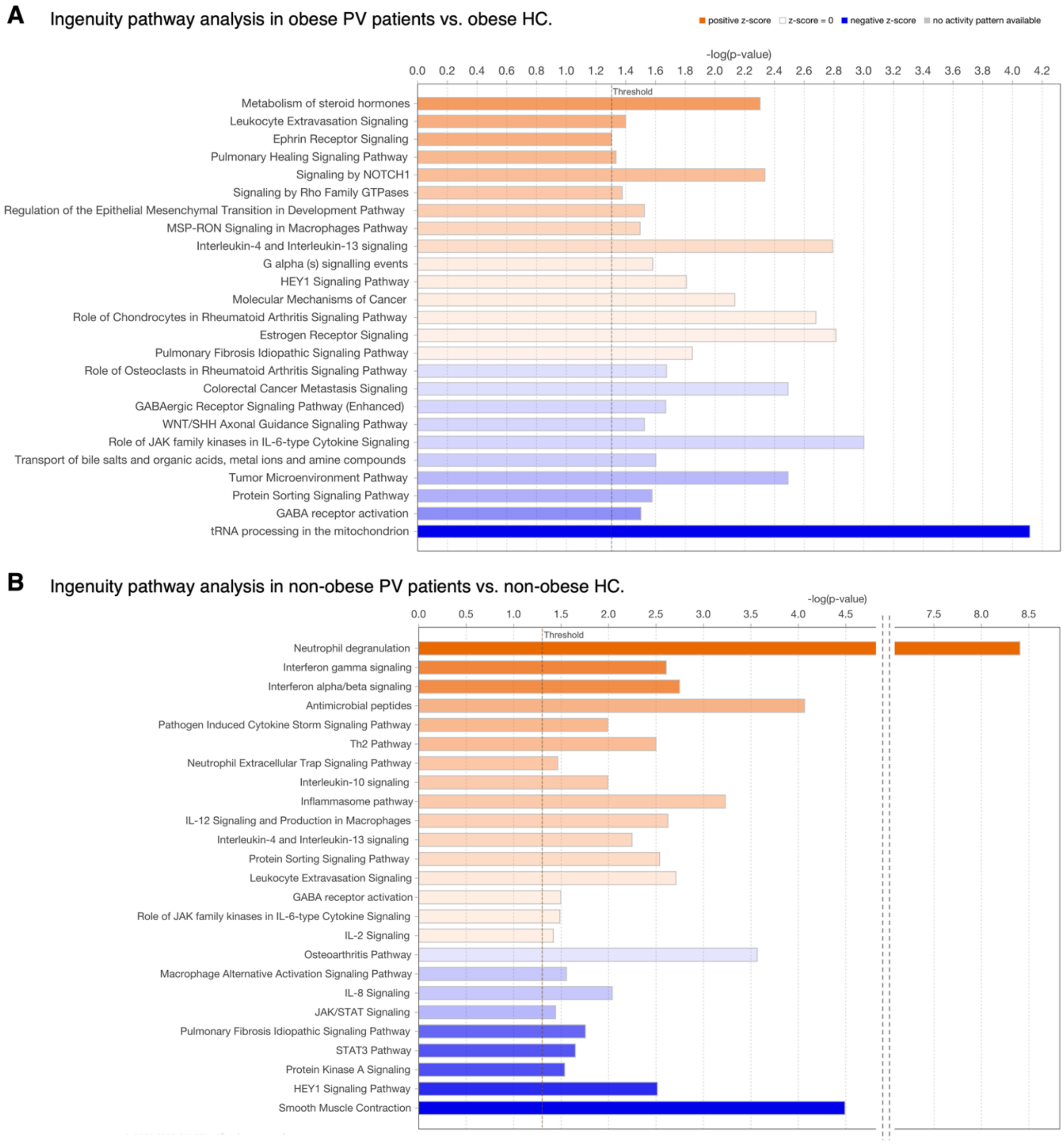

Supplementary Figure 5. Enrichment pathways identified by ingenuity pathway analysis comparing psoriasis patients and healthy controls by body weight. (A) obese PV vs. obese HC and (B) non-obese PV vs. non-obese HC. PV, psoriasis vulgaris; HC, healthy control.

Gene clusters that compose the enrichment pathway in obese PV patients vs. obese HC.

Ingenuity Canonical Pathways	-log (p-value)	Ratio	z-score	Genes
Metabolism of steroid hormones	1.64	0.114	2	HSD17B2,POMC,TSPO,TSPOAP1
Leukocyte Extravasation Signaling	0.594	0.0415	1.633	ARHGAP5,BCAR1,CLDN23,CLDN7,MMP11,MMP17,MMP23B,PIK3C2A
Ephrin Receptor Signaling	1.28	0.0545	1.633	ANGPT1,BCAR1,EPHA10,EPHB3,GNB1L,GNG10,GNG2,GRIN1,ITGB4,PAK4,PROK1
Pulmonary Healing Signaling Pathway	1.03	0.0503	1.897	DLK1,FZD1,FZD2,MMP11,MMP17,MMP23B,NGFR,PROK1,RBPJ,WNT6
Signaling by NOTCH1	1.46	0.0769	1.342	DLK1,DLL1,JAG2,MIB2,RBPJ,TBL1XR1
Signaling by Rho Family GTPases	0.881	0.0449	1.414	ARHGEF16,BAIAP2,CDC42EP5,CDH3,GNB1L,GNG10,GNG2,ITGB4,MYL11,PAK4,PIK3C2A,RHOQ
Regulation of the Epithelial Mesenchymal Transition in Development Pathway	1.27	0.069	1.342	FZD1,FZD2,JAG2,PYGO1,RBPJ,WNT6
MSP-RON Signaling in Macrophages Pathway	0.776	0.0504	0.816	F12,HLA-DQB2,KLK10,KLK14,PIK3C2A,SBNO2
Interleukin-4 and Interleukin-13 signaling	3.19	0.1	0.905	CD36,CEBPD,FGF2,FSCN1,IGHG4,IL13,JUNB,LAMA5,LCN2,MAOA,POMC
G alpha (s) signaling events	3.21	0.0897	0.277	ADCYAP1,CALCR,CRHR2,GCGR,GLP1R,GNG10,GNG2,PDE11A,PDE3B,PDE8B,POMC,RAMP1,VIP
HEY1 Signaling Pathway	2.79	0.0807	0	ACVR1C,BMP3,BMP5,BMP8A,DLL1,E2F2,JAG2,MMP11,MMP17,MMP23B,PIK3C2A,PROK1,RBPJ
Molecular Mechanisms of Cancer	2.82	0.0502	0.152	ADGRB1,APC2,ARHGEF16,BAD,BMP3,BMP5,BMP8A,CALCR,CELSR1,CRHR2,DHH,E2F2,F2RL2,FGF2,FZD1,FZD2,GCGR,GHR,GLP1R,GNB1L,GNG10,GNG2,GPR158,GPR180, GPR34,HCAR1,IL1RL2,IL6ST,IL9R,ITGB4,MMP11,MMP17,MMP23B,NPY5R,OPRK1,PAK4,PIK3C2A,PRKAR2B,PTGER3,RBPJ,RHOQ,S1PR4,SUCNR1
Role of Chondrocytes in Rheumatoid Arthritis Signaling Pathway	1.89	0.0709	0.632	CEBPD,GNB1L,IL1RL2,IL6ST,MMP11,MMP17,MMP23B,NGFR,PPP3R1,PROK1
Estrogen Receptor Signaling	2.61	0.0587	1	ATP5F1D,BAD,CACNA1B,CACNA1D,CACNA2D1,CAV1,GNB1L,GNG10,GNG2,LEPR,MED16,MMP11,MMP17,MMP23B,MYL11,NDUFB7,NDUFS7,NRIP1,PIK3C2A,PLCH1,PLCH2, PRKAR2B,PROK1,TBL1XR1
Pulmonary Fibrosis Idiopathic Signaling Pathway	2.15	0.0583	1.147	ACVR1C,CAV1,CERT1,COL26A1,COL7A1,COL9A2,COL9A3,EPHB3,FGF2,FZD1,FZD2,IL13,IL6ST,MMP11,MMP17, MMP23B,PIK3C2A,RBPJ,WNT6
Role of Osteoclasts in Rheumatoid Arthritis Signaling Pathway	1.77	0.0552	1	ADAM22,BAD,CALCR,CALML5,CERT1,COL26A1,COL7A1,COL9A2,COL9A3,IL1RL2,MMP11,MMP17,MMP23B,NGFR,PIK3C2A,PPP3R1,RHOQ
Colorectal Cancer Metastasis Signaling	1.95	0.059	-0.535	BAD,FZD1,FZD2,GNB1L,GNG10,GNG2,IL6ST,MMP11,MMP17,MMP23B,PIK3C2A,PRKAR2B,PROK1,PTGER3,RHOQ,WNT6
GABAergic Receptor Signaling Pathway (Enhanced)	2.37	0.0791	-0.905	CACNA1B,CACNA1D,CACNA1G,CACNA2D1,GABRA2,GABRB2,GNB1L,GNG10,GNG2,GRIN1,PRKAR2B
WNT/SHH Axonal Guidance Signaling Pathway	1.36	0.0604	0.333	FZD1,FZD2,HHIP,PIK3C2A,PRKAR2B,SEMA3A,SEMA3D,SFN,WNT6
Role of JAK family kinases in IL-6-type Cytokine Signaling	1.94	0.0886	-0.378	CEBPD,IL6ST,JUNB,LIFR,POMC,PROK1,VIP
Transport of bile salts and organic acids, metal ions and amine compounds	2.37	0.0964	-0.707	SLC16A7,SLC18A2,SLC22A18,SLC22A4,SLC2A13,SLC39A4,SLC47A1,SLC6A15
Tumor Microenvironment Pathway	1.98	0.067	-0.577	BAD,FGF2,IL13,LEPR,MMP11,MMP17,MMP23B,PIK3C2A,PROK1,SLC2A1,TNC,TSLP
Protein Sorting Signaling Pathway	0.978	0.0506	-1	COL7A1,PRKAR2B,SNX10,SNX16,SNX22,SNX32,TMED5,TMED7,WASHC4
GABA receptor activation	0.933	0.0667	-1	GABRA2,GABRB2,GNG10,GNG2
tRNA processing in the mitochondrion	3.21	0.217	-2.236	MT-TD,MT-TI,MT-TL1,MT-TM,MT-TV

Supplementary Figure 6. The gene clusters composing the enrichment pathways in obese PV vs. obese HC (Supplementary Figure 5A). PV, psoriasis vulgaris; HC, healthy control.

Gene clusters that compose the enrichment pathway in non-obese PV patients vs. non-obese HC.

Ingenuity Canonical Pathways	-log (p-value)	Ratio	z-score	Genes
Neutrophil degranulation	8.41	0.0839	3.683	ACLY,ADAM8,ADGRG3,AGL,ARG1,ARHGAP45,ATP8A1,CEACAM3,CLEC4D,CXCL1,ENPP4,FABP5,FCGR3B,FCN1,FPR2,FRK,ITGAV,ITGB2,MCEMP1,MGAM,MMP25,MS4A3,ORM1, PGLYRP1,PTAFR,PTPRJ,RAB24,RAP1A,RNASE2,S100A12,S100A7,S100A8,S100A9,SCAMP1,SERPINA1,SERPINB3,SIRPB1,TIMP2,TLR2,TXNDC5
Interferon gamma signaling	2.61	0.0918	3	CIITA,GBP5,HLA-H,IFI30,IRF7,OAS2,OAS3,OASL,PTAFR
Interferon alpha/beta signaling	2.75	0.105	2.828	HLA-H,IFI6,IRF7,ISG15,OAS2,OAS3,OASL,RSAD2
Antimicrobial peptides	4.07	0.163	1.89	ITLN1,PGLYRP1,PGLYRP2,PI3,S100A7,S100A7A,S100A8,S100A9
Pathogen Induced Cytokine Storm Signaling Pathway	1.99	0.0524	1.789	CIITA,COL20A1,COL4A3,CXCL1,CXCR4,FOS,HLA-DOB,IL12RB2,IL1B,IL6ST,IRF7,LTB,MAPK4,NLRP12,NOD2,P2RX7,RYR2,STXBP2,TLR2,TLR8
Th2 Pathway	2.5	0.078	1.667	BHLHE41,BMPR2,CXCR4,CXCR6,HLA-DOB,IL12RB2,ITGB2,JAK3,PIK3C2A,TGFBR3,TNFRSF4
Neutrophil Extracellular Trap Signaling Pathway	1.46	0.0467	1.414	BLK,CASP12,CASP5,COL20A1,COL4A3,FCGR3B,FRK,IL1B,ITGB2,LCK,MT-ND6,NDUFA5,PADI4,PIK3C2A,PLB1,PLCB4,PPP3CA,TLR2,TLR8
Interleukin-10 signaling	1.99	0.111	1.342	CCR2,CXCL1,FCER2,IL1B,PTAFR
Inflammasome pathway	3.23	0.208	1.342	CASP5,IL1B,NEK7,NOD2,P2RX7
IL-12 Signaling and Production in Macrophages	2.63	0.0661	1.155	ALOX15,APOD,FCGR3B,FOS,FUT7,IL12RB2,IL6ST,LCK,NOD2,ORM1,PIK3C2A,PRKAA2,PRL,S100A8,SERPINA1, TLR2
Interleukin-4 and Interleukin-13 signaling	2.25	0.0811	1	ALOX15,BIRC5,FCER2,FGF2,FOS,IL1B,ITGB2,JAK3,POMC
Protein Sorting Signaling Pathway	2.54	0.0718	0.905	AP1S2,BLK,FRK,GNAQ,LCK,MIA2,PLD5,RAB11FIP1,SNX20,SNX22,SNX32,TMED7,WASHC2C
Leukocyte Extravasation Signaling	2.71	0.0722	0.577	ACTA2,ARHGAP5,CLDN11,CLDN23,CTNNA3,CXCR4,EDIL3,ICAM3,ITGB2,MMP16,MMP25,PIK3C2A,RAP1A,TIMP2
GABA receptor activation	1.5	0.0833	0.447	GABRA2,GABRB2,GNGT2,KCNJ15,KCNJ2
Role of JAK family kinases in IL-6-type Cytokine Signaling	1.49	0.0741	0.447	BIRC5,FOS,IL6ST,LIFR,POMC,SERPINA1
IL-2 Signaling	1.42	0.0794	0.447	FOS,JAK3,LCK,PIK3C2A,RAP1A
Osteoarthritis Pathway	3.57	0.0756	-0.302	ACAN,ADAMTS5,BMPR2,CASP12,CASP5,FGF2,IL1B,ITGAV,ITGB2,ITGB8,ITLN1,P2RX7,PRKAA2,RBPJ,S100A8,S100A9,SMAD9,TLR2
Macrophage Alternative Activation Signaling Pathway	1.56	0.0573	-0.632	ABCA1,ACLY,ALOX15,ARG1,CIITA,FCER2,FOS,HLA-DOB,IL1B,JAK3,PIK3C2A
IL-8 Signaling	2.04	0.0625	-0.632	CCND2,CXCL1,FOS,GNAQ,HBEGF,ITGAV,ITGB2,MYL9,PIK3C2A,PLD5,RAB11FIP2,RAP1A,RHOQ
JAK/STAT Signaling	1.44	0.0723	-0.816	CISH,FOS,GNAQ,JAK3,PIK3C2A,RAP1A
Pulmonary Fibrosis Idiopathic Signaling Pathway	1.76	0.0521	-1.698	ACTA2,BIRC5,BMPR2,CAV1,COL20A1,COL4A3,FGF2,FOS,IL1B,IL6ST,ITGAV,MMP16,MMP25,PIK3C2A,RAP1A,RBPJ,TGFBR3
STAT3 Pathway	1.65	0.0647	-1.89	BMPR1B,BMPR2,CISH,FGF2,IL12RB2,IL1B,IL6ST,RAP1A,TGFBR3
Protein Kinase A Signaling	1.54	0.0476	-1.941	AKAP11,AKAP3,DUSP16,FLNA,FLNC,GNAQ,MTMR7,MYL9,NTN1,PDE4C,PDE5A,PLCB4,PLN,PPP1CB,PPP3CA,PTPRJ,PTPRR,RAP1A,RYR2
HEY1 Signaling Pathway	2.51	0.0745	-2.309	BMP3,BMPR1B,BMPR2,ERBB4,MMP16,MMP25,MYH11,PIK3C2A,PRKAA2,RBPJ,SMAD9,TGFBR3
Smooth Muscle Contraction	4.49	0.186	-2.828	ACTA2,CALD1,GUCY1A2,LMOD1,MYH11,MYL9,PDE5A,TPM1

Supplementary Figure 7. The gene clusters composing the enrichment pathways in non-obese PV vs. non-obese HC (Supplementary Figure 5B). PV, psoriasis vulgaris; HC, healthy control.

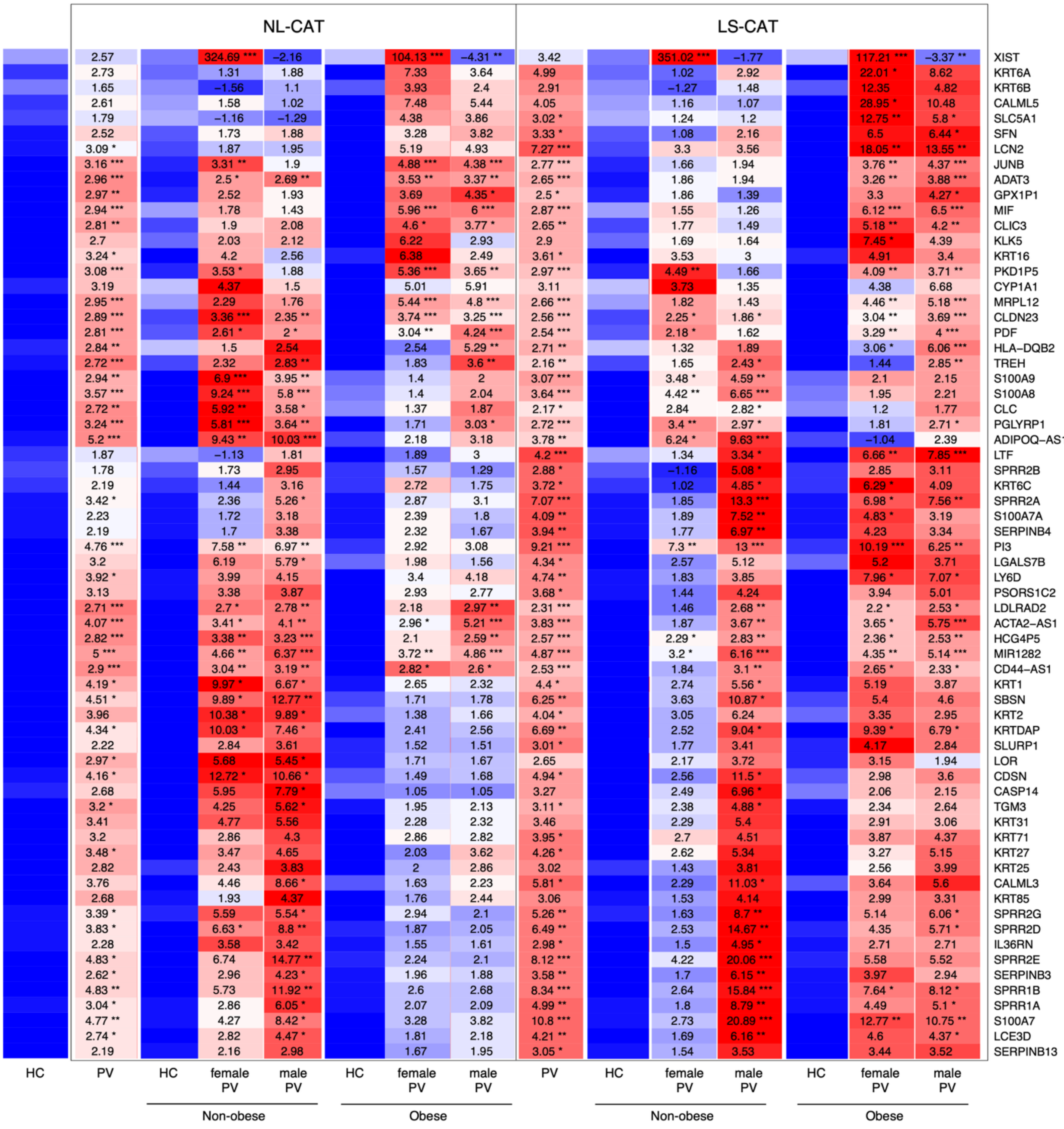

Supplementary Figure 8. Comparison of top upregulated genes in baseline by sex between obese and non-obese groups. *, **, *** indicate $p < 0.05$, $p < 0.01$, $p < 0.001$, respectively. PV, psoriasis vulgaris; HC, healthy control; LS-CAT, lesional skin cutaneous adipose tissue; NL-CAT, non-lesional skin cutaneous adipose tissue.

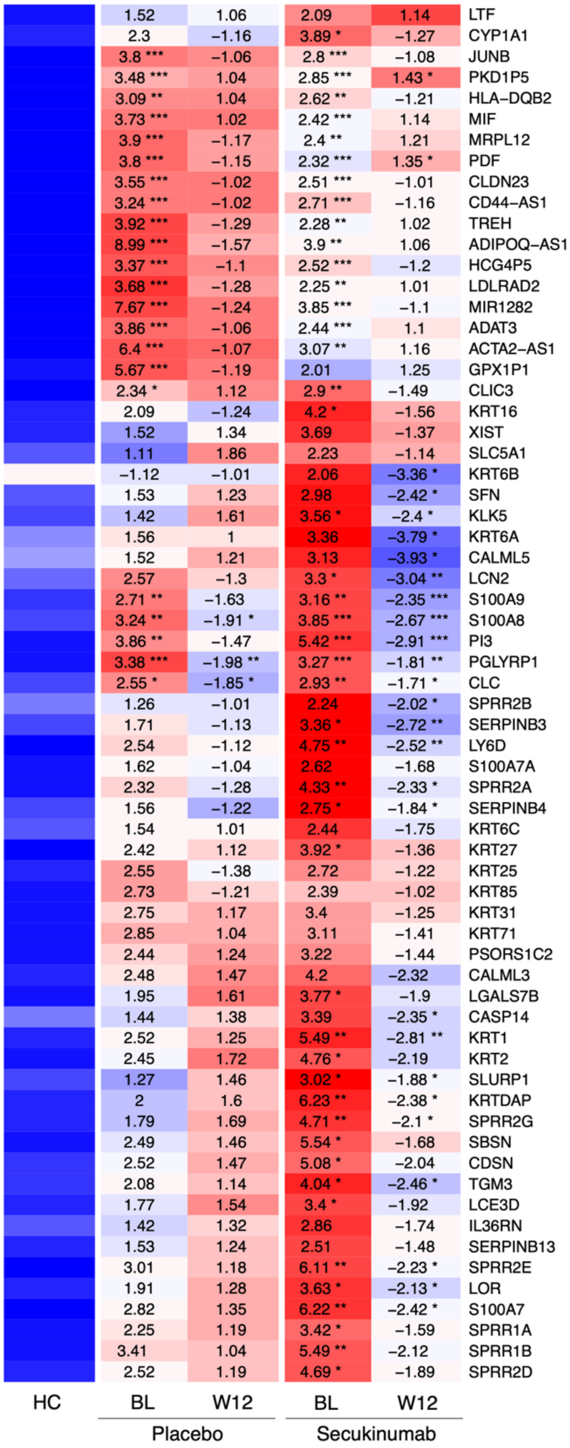

Supplementary Figure 9. The top upregulated genes of baseline NL-CAT in placebo vs. secukinumab treatment groups. *, **, *** indicate $p < 0.05$, $p < 0.01$, $p < 0.001$, respectively. HC, healthy control; BL, baseline.

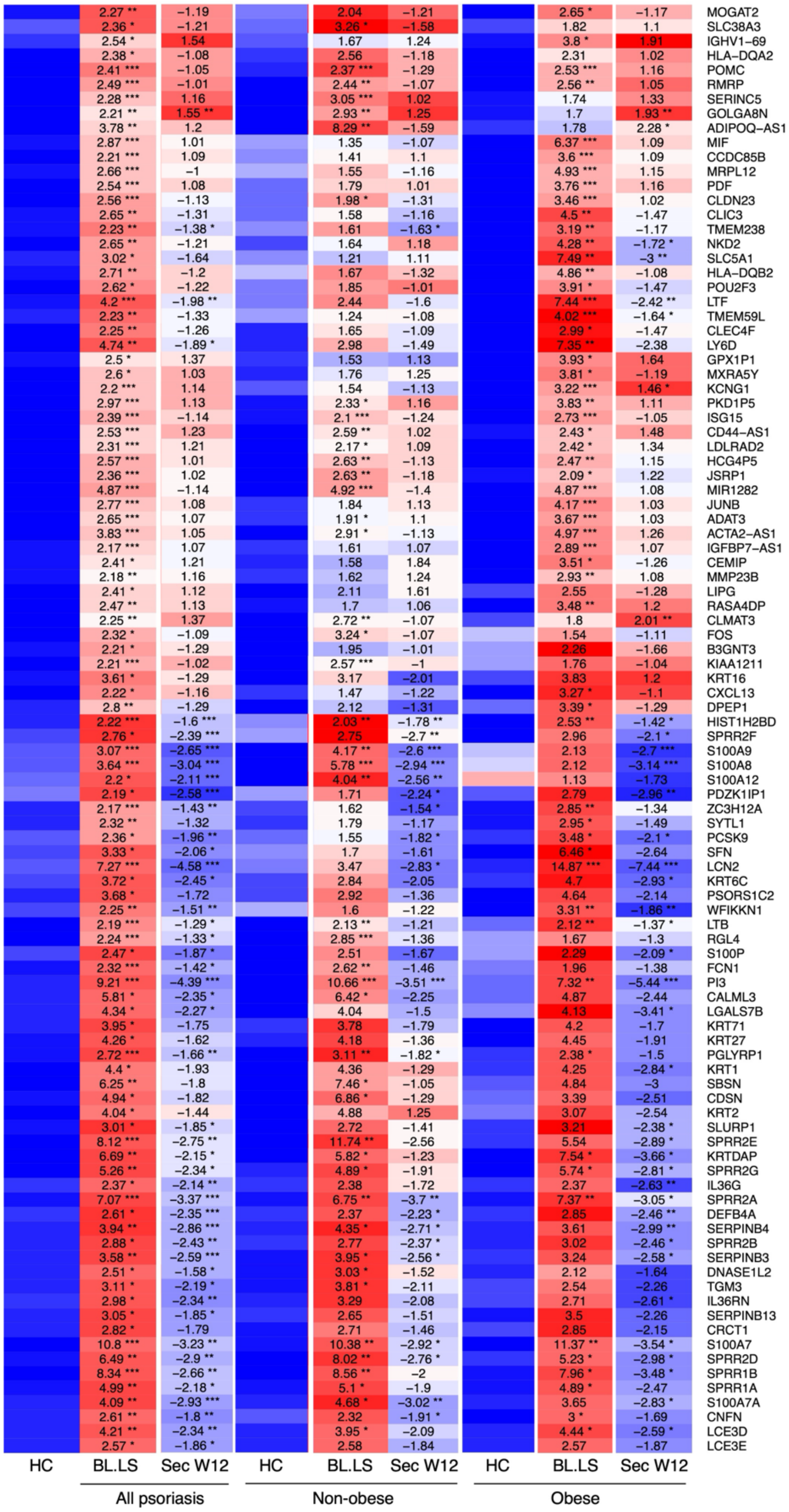

Supplementary Figure 10. Comparison of improvement in top upregulated transcripts in baseline LS-CAT by body weight group following secukinumab treatment. *, **, *** indicate $p < 0.05$, $p < 0.01$, $p < 0.001$,

respectively. HC, healthy control; BL.LS, baseline lesional skin cutaneous adipose tissue; Sec W12, secukinumab treatment at week 12.

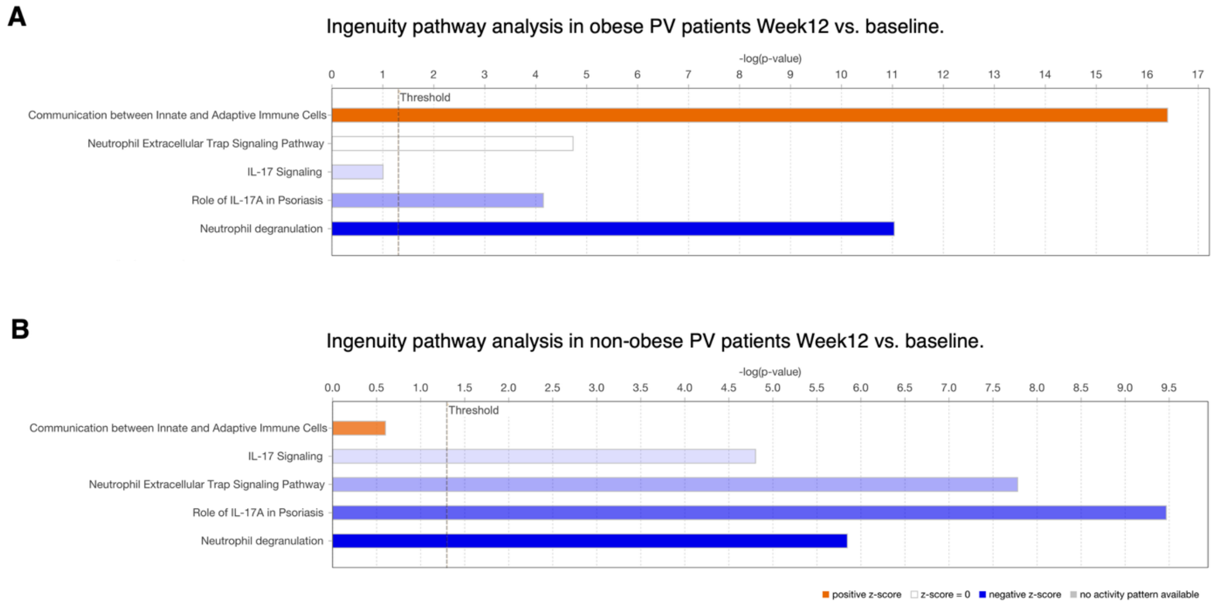

Supplementary Figure 11. Pathways most altered by secukinumab treatment in (A) obese and (B) non-obese psoriasis patients using ingenuity pathway analysis. PV, psoriasis vulgaris.

A. Gene clusters that compose the enrichment pathway in obese PV patients Week 12 vs. baseline.

Ingenuity Canonical Pathways	-log (p-value)	Ratio	z-score	Genes
Communication between Innate and Adaptive Immune Cells	16.4	0.0612	6.682	IGHA1,IGHG2,IGHG4,IGHJ5,IGHV1-18,IGHV1-2,IGHV1-24,IGHV1-46,IGHV3-11,IGHV3-15,IGHV3-33,IGHV3-43,IGHV3-49,IGHV3-72,IGHV4-34,IGHV4-39,IGHV4-61,IGHV5-51,IGKV1-16,IGKV1-17,IGKV1-33,IGKV1-6,IGKV1-9,IGKV1D-16,IGKV2-30,IGKV2D-29,IGKV2D-40,IGKV3-11,IGKV3-15,IGKV3-20,IGKV3D-15,IGKV3D-20,IGLC2,IGLC3,IGLC7,IGLV1-36,IGLV1-40,IGLV1-44,IGLV1-51,IGLV2-14,IGLV2-18,IGLV3-10,IGLV3-19,IGLV3-21,IGLV3-25,IGLV3-9,IGLV4-69,IGLV5-45,IGLV6-57,IGLV7-43,IGLV7-46,IGLV9-49,IL1B,IL36G,IL36RN,JCHAIN,TRBV20-1
Neutrophil Extracellular Trap Signaling Pathway	4.73	0.05	0	CASP5,COL11A1,COL9A3,CXCR2,FCGR3A/FCGR3B,FPR1,IGHA1,IGHG2,IGHG4,IGLC2,IGLC3,IGLC7,IL1B,JCHAIN,LTF,PADI4,PLA2G2A,PLA2G2D,PLA2G3,PLA2G4E
IL-17 Signaling	1	0.0321	-0.816	DEFB4A/DEFB4B,IL13,IL1B,IL36G,LCN2,MMP13
Role of IL-17A in Psoriasis	4.15	0.286	-2	DEFB4A/DEFB4B,S100A7,S100A8,S100A9
Neutrophil degranulation	11	0.0693	-5.396	ADGRE3,ADGRG3,AOC1,ARG1,CALML5,CDA,CEACAM3,CRISP3,CXCR1,CXCR2,DSG1,FCGR3A/FCGR3B,FPR1,FPR2,HBB,KRT1,LCN2,LTF,MGAM,MME,MMP25,OLFM4,PADI2,PRSS3,S100A7,S100A8,S100A9,S100P,SELL,SERPINA1,SERPINB3,SLPI,TCN1

B. Gene clusters that compose the enrichment pathway in non-obese PV patients Week 12 vs. baseline.

Ingenuity Canonical Pathways	-log (p-value)	Ratio	z-score	Genes
Communication between Innate and Adaptive Immune Cells	0.601	0.0172	3	CXCL8,IGHA1,IGHG1,IGHG2,IGHJ6,IGHV1-2,IGHV4-59,IGKV1-39,IGKV1-6,IGKV3-20,IGLC2,IGLC7,IGLV3-9,IGLV7-43,IL1B,JCHAIN
IL-17 Signaling	4.8	0.0642	-0.577	CXCL1,CXCL5,CXCL8,DEFB124,DEFB4A/DEFB4B,IL1B,LCN2,MMP2,PGF,PROK1,RGS16,TGFB3
Neutrophil Extracellular Trap Signaling Pathway	7.78	0.0575	-1.46	CASP5,COL10A1,COL1A1,COL1A2,COL20A1,COL21A1,COL3A1,COL5A1,COL5A2,COL6A3,CXCL8,CXCR2,FCGR3A/FCGR3B,FPR1,IGHA1, IGHG1,IGHG2,IGLC2,IGLC7,IL1B,JCHAIN,MMP2,PADI4
Role of IL-17A in Psoriasis	9.47	0.5	-2.646	CXCL1,CXCL5,CXCL8,DEFB4A/DEFB4B,S100A7,S100A8,S100A9
Neutrophil degranulation	5.85	0.0462	-4.264	ADGRE3,ADGRG3,CEACAM3,CXCL1,CXCR1,CXCR2,FCGR3A/FCGR3B,FPR1,FPR2,LCN2,LRG1,MGAM,MMP25,PGLYRP1,S100A12,S100A7, S100A8,S100A9,SERPINA1,SERPINB3,SLC27A2,TNFAIP6

Supplementary Figure 12. The gene clusters composing the enrichment pathways in (A) obese PV patients week 12 vs. baseline and (B) non-obese PV patients week 12 vs. baseline (Supplementary Figure 11). PV, psoriasis vulgaris.

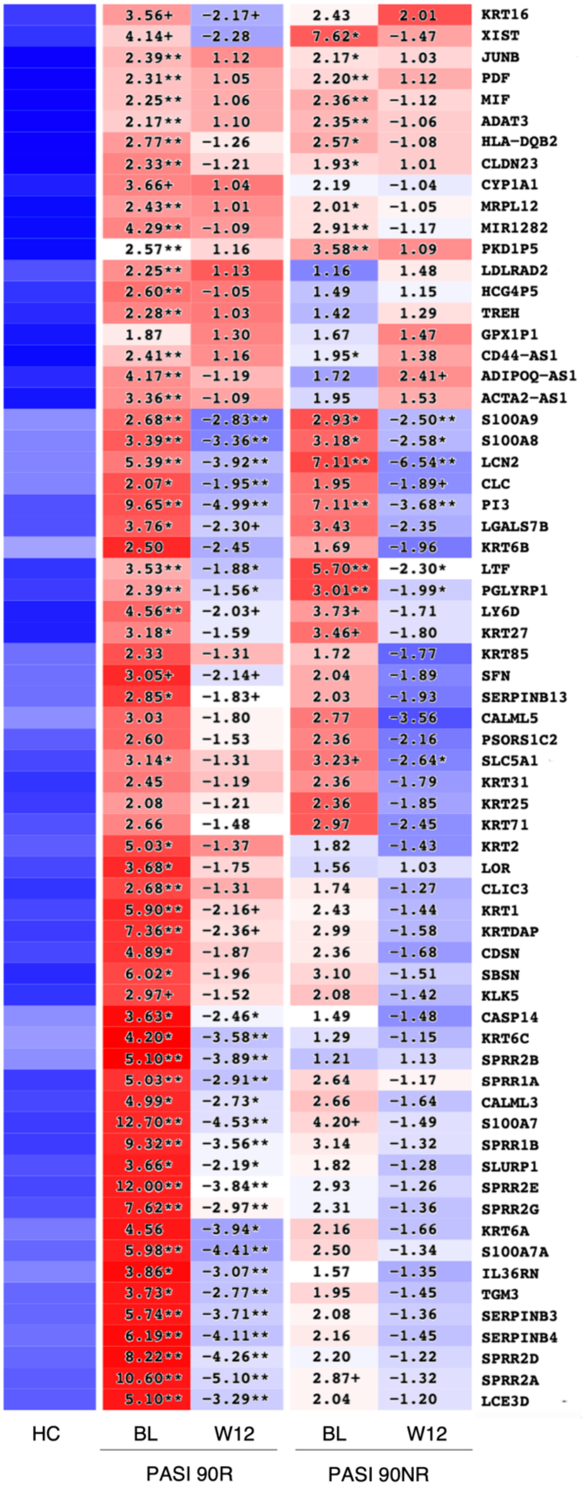

Supplementary Figure 13. Top expressed genes of psoriatic CAT in PASI 90R and PASI 90NR. PASI, psoriasis area and severity index; PASI 90R, PASI 90 responders; PASI 90NR, PASI 90 non-responders; BL, baseline. *, **, *** indicate $p < 0.05$, $p < 0.01$, $p < 0.001$, respectively.

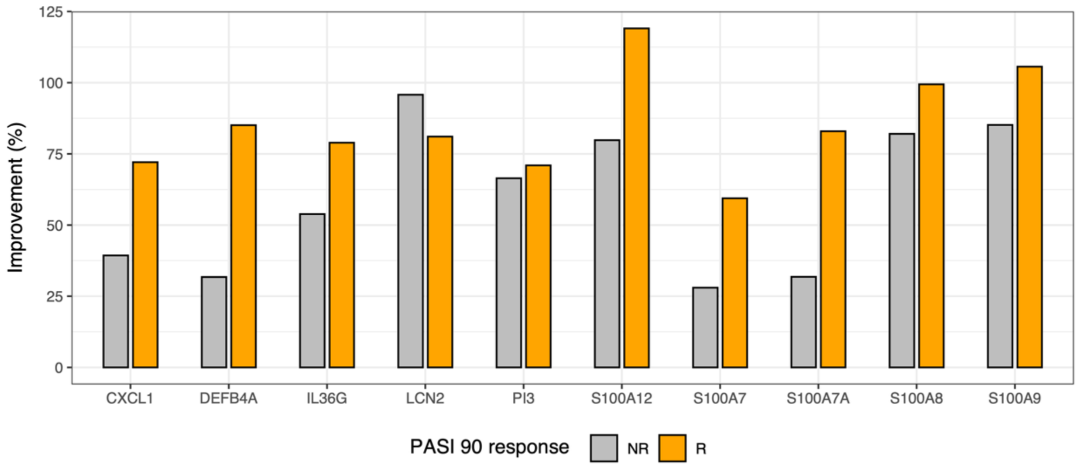

Supplementary Figure 14. Comparison of improvement in PASI 90R and PASI 90NR of essential genes for psoriatic CAT. PASI, psoriasis area and severity index; PASI 90R, PASI 90 responders; PASI 90NR, PASI 90 non-responders; BL, baseline.

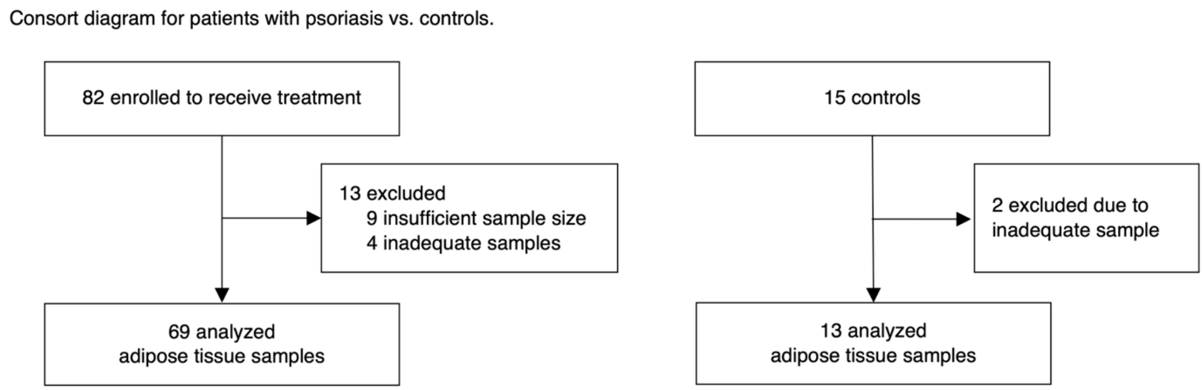

Supplementary Figure 15. Consort diagram for patients with psoriasis vs. controls.

Consort diagram for treatment with secukinumab or placebo.

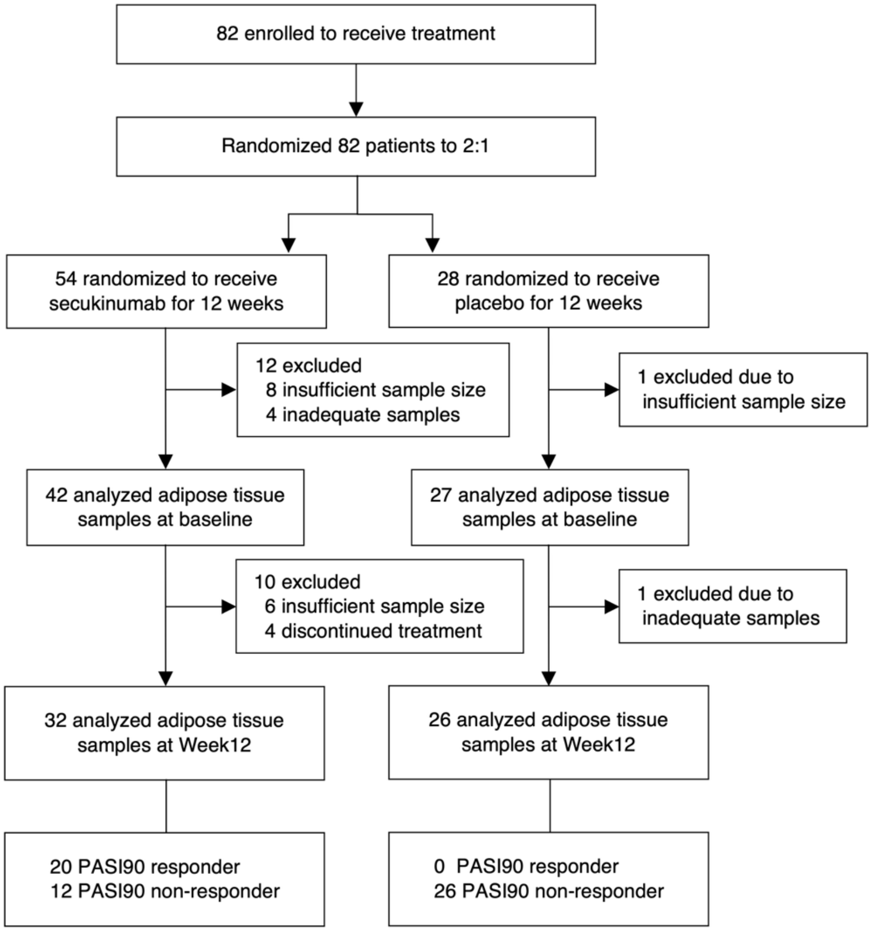

Supplementary Figure 16. Consort diagram for treatment with secukinumab or placebo.
